# Child Deaths Due to Injury in the Four UK Countries: A Time Trends Study from 1980 to 2010

**DOI:** 10.1371/journal.pone.0068323

**Published:** 2013-07-10

**Authors:** Pia Hardelid, Jonathan Davey, Nirupa Dattani, Ruth Gilbert, the Working Group of the Research and Policy Directorate of the Royal College of Paediatrics and Child Health

**Affiliations:** Centre for Paediatric Epidemiology and Biostatistics, University College London Institute of Child Health, London, United Kingdom; University of Cincinnati, United States of America

## Abstract

**Background:**

Injuries are an increasingly important cause of death in children worldwide, yet injury mortality is highly preventable. Determining patterns and trends in child injury mortality can identify groups at particularly high risk. We compare trends in child deaths due to injury in four UK countries, between 1980 and 2010.

**Methods:**

We obtained information from death certificates on all deaths occurring between 1980 and 2010 in children aged 28 days to 18 years and resident in England, Scotland, Wales or Northern Ireland. Injury deaths were defined by an external cause code recorded as the underlying cause of death. Injury mortality rates were analysed by type of injury, country of residence, age group, sex and time period.

**Results:**

Child mortality due to injury has declined in all countries of the UK. England consistently experienced the lowest mortality rate throughout the study period. For children aged 10 to 18 years, differences between countries in mortality rates increased during the study period. Inter-country differences were largest for boys aged 10 to 18 years with mortality rate ratios of 1.38 (95% confidence interval 1.16, 1.64) for Wales, 1.68 (1.48, 1.91) for Scotland and 1.81 (1.50, 2.18) for Northern Ireland compared with England (the baseline) in 2006–10. The decline in mortality due to injury was accounted for by a decline in unintentional injuries. For older children, no declines were observed for deaths caused by self-harm, by assault or from undetermined intent in any UK country.

**Conclusion:**

Whilst child deaths from injury have declined in all four UK countries, substantial differences in mortality rates remain between countries, particularly for older boys. This group stands to gain most from policy interventions to reduce deaths from injury in children.

## Introduction

Prevention of deaths in children is a key aim of public health policy at national and international level, and child mortality is a widely used indicator of economic and social development [1]. A recent comparative study of child and adolescent mortality in developed countries showed that whilst mortality rates in younger children have declined since the 1950s, reductions in mortality for older children and adolescents have been much less marked [2]. This study identified injuries as an increasingly important cause of death in children globally, as mortality rates due to infections and chronic diseases decline.

Deaths due to injuries are highly preventable [3]. Information on trends and patterns of injury deaths can guide targeting of preventive polices at groups with the highest levels of preventable deaths. The risk of death due to injury is higher in boys than in girls [4], and in children whose parents have low socio-economic status[5]–[7], highlighting the importance of social factors as risk factors for these deaths. Inter-country variation in deaths due to injury in Scandinavian countries with similar socioeconomic profiles and welfare systems may reflect the additional effect of public health policy on child injury deaths [8].

In this study, we compare time trends in child mortality rates due to injuries in four countries of the UK, by age group and sex. We hypothesise that variation between countries may reflect the impact of policies such as health promotion programmes for injury reduction, healthcare management of serious injury, or public health interventions to reduce social, economic or neighbourhood determinants of injury. The results should inform preventive policies by identifying groups most likely to benefit.

## Methods

### Ethics Statement

All data were anonymised and no individual could be identified. Therefore ethical approval was not sought.

We used anonymised death registration data for deaths occurring between January 1980 and December 2010 in children aged 28 days to 18 years inclusive. Data were obtained from national statistics agencies in the four UK countries: the Office for National Statistics (ONS-covering deaths registered in England and Wales) and the Northern Ireland Statistics and Research Agency (NISRA). For Scotland, death certificate data collected and coded by National Records for Scotland were obtained through the Information Services Division (ISD) of National Health Service Scotland. The data extracts included deaths registered until 27^th^ June 2012 for England and Wales, 3^rd^ May 2012 for Scotland and 18^th^ June 2012 for Northern Ireland. We excluded neonatal deaths (before 28 days of age) because death certificates do not provide a single underlying cause of death for this group. We also excluded children who were not resident in the country where they died.

We defined a death as due to injury, (including poisoning), if the underlying cause of death used an external cause code from the International Classification of Diseases (ICD) [9]. The ICD revision 9 external cause codes (E800–E999) were used between 1980 and 2000 in England, Wales and Northern Ireland and between 1980 and 1999 in Scotland. Revision 10 external cause codes (V01–Y89) were used after this. We identified unintentional injuries due to transport, other injuries and complications of medical and surgical care using ICD-9 codes E800–E949 and ICD-10 codes V01-X59, Y40–Y86, Y88. Intentional injuries (chiefly assault, self-harm and events of undetermined intent) were identified using ICD-9 codes E950–E989 and ICD-10 codes X60-Y36, Y87, Y89, U50.9 [10].

Age at death was coded into a two category variable (28 days to 9 years, and 10 to 18 years) to ensure sufficient number of deaths for meaningful analyses. Denominator populations by age group, sex and year were obtained from the ONS, ISD and NISRA. For England and Wales, denominator populations for the years 2001 to 2010 had been updated following the 2011 census; updated denominators were not yet available for Scotland and Northern Ireland.

We analysed deaths by year of occurrence. We initially adjusted mortality rates for England, Wales and Northern Ireland to account for reporting delay. Adjustment was not required for Scotland, since deaths referred to the Procurator Fiscal in Scotland can still be registered without waiting for the outcome of a possible inquest [11]. Updated mortality data for deaths occurring in the study period but registered in the first two quarters of 2012 subsequently became available. Following these updates, further adjustments for reporting delays made little difference to mortality rates, and unadjusted rates were therefore used. Our methods initially used for reporting delay adjustment are presented as Supporting Information (Text S1 and Figure S1). We visually examined the mortality time series for spurious change points due to the change in the ICD version, but no apparent interruptions were found.

To create plots of mortality time series, we calculated three year moving averages of annual mortality rates to smooth large variations in annual rates due to the small number of deaths. Due to the small number of events per year in Scotland, Wales and Northern Ireland, the study period was split a priori into three five-year time periods from each decade of the study period (1980–84, 1993–97 and 2006–10) to allow for inter-country comparisons of time trends. We stratified rates per 100,000 population by age group, sex, country and time period and calculated 95% confidence intervals (CIs). We compared countries by calculating rate ratios and rate differences for Scotland, Wales and Northern Ireland compared with England (the baseline rate). We calculated population attributable risks for observed inter-country differences for the period 2006–2010 by applying the point estimate of the age group and sex specific risk difference between England and Scotland, Wales and Northern Ireland to the population by age group and sex of these three countries respectively. Population attributable risks were only calculated if significant differences in rates were observed.

To examine whether mortality time trends varied by country, we fitted Poisson regression models to counts of deaths with sex, country and time period as covariates. A country:time period interaction term was then added to examine whether this significantly improved the model fit. Models were fitted separately to each age group. For the older age group, models were fitted using a quasi-likelihood method to take overdispersion into account [12]. Likelihood ratio (LR) tests (for the youngest age group) and *F*-tests (for the oldest age group) were used to determine whether the addition of further parameters significantly improved model fit. Denominator populations were included as an offset in the models.

We examined trends in unintentional and intentional injury by plotting three-year rolling averages of mortality rates by year of death. We further subdivided the unintentional injury category into transport injuries and other unintentional injuries (including misadventures of medical and surgical care) and calculated mortality rates by type of injury, country and time period. For England and Wales, a large number of injuries caused by motor vehicle traffic were coded as ICD9 code E928 (other and unspecified environmental and accidental causes) between 1991 and 1995 (personal communication with ONS Mortality Statistics Team, 20^th^ September 2012). A spurious decline in transport injuries, and a concurrent spurious increase in other accidental injuries were consequently observed for England and Wales for the middle time period (1993–1997). We therefore did not calculate rates for the two accident types for this time period for England and Wales. This coding inconsistency does not affect the overall mortality rate for unintentional injuries. We present combined analyses for boys and girls to avoid small cell sizes. All analyses were carried out using Stata 12.1 [13] and R 12.15.2 [14].

## Results

**Figure 1:**
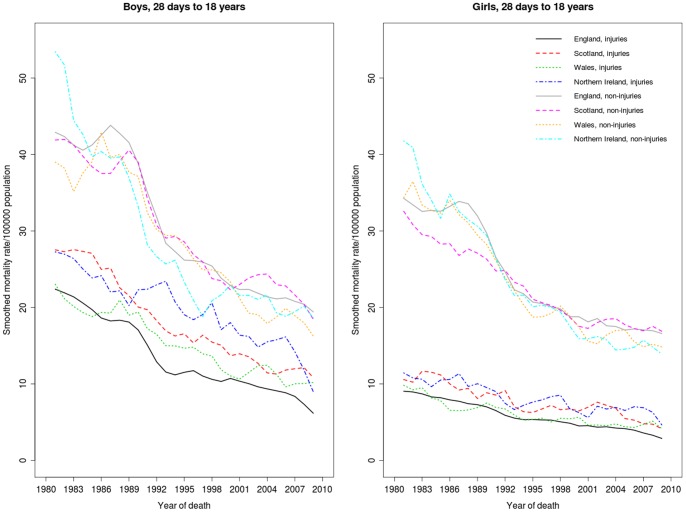
Mortality rates (three-year moving averages) for children aged 28 days to 18 years by country, gender and type of cause (injury or other cause), by year of death, 1980–2010. Note the three-year average rates are plotted on the central year.

The study comprised 136,794 children who died in England, 15,428 in Scotland, 8,111 in Wales and 6,271 in Northern Ireland between 1980 and 2010. These numbers exclude 3,138, 348 and 31 non-resident children in England and Wales, Scotland and Northern Ireland respectively, as well as four children in England and Wales for whom the year of death was missing. Figure 1 shows smoothed mortality rates by sex and country for deaths due to injury and other causes. Injury mortality rates declined by between 50% and 70% for both boys and girls in the study period; similar declines were also observed for other causes of death.

**Figure 2:**
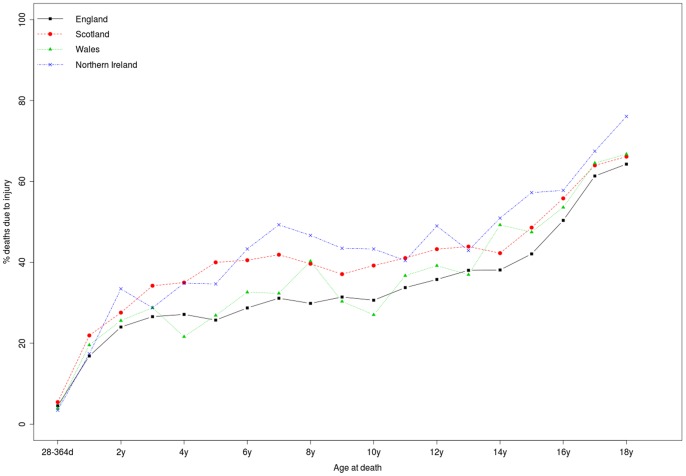
Percentage of child deaths due to injury by country and age at death, 1980–2010.

Across the entire age range (28 days to 18 years), deaths from injury constituted the largest single underlying cause of death of all the ICD chapters in the study period, accounting for 36,060/136,794 (26.4%) child deaths in England, 5,102/15,428 (33.1%) in Scotland, 2,393/8,111 (29.5%) in Wales and 2,148/6,271 (34.3%) in Northern Ireland. Figure 2 shows that the proportion of child deaths due to injury increased with age in all four countries.

Transport accidents accounted for 47.3% of deaths due to injury (17,067/36,060 deaths) in England, 46.7% in Scotland (2,382/5,102), 41.3% in Wales (989/2,393), and 52.4% in Northern Ireland (1,126/2,148).

Considering point estimates of mortality rates, England has the lowest mortality due to injury among all countries by age and sex in the majority of time periods, as shown Table 1. However, mortality rate point estimates for all countries apart from England have wide 95% confidence intervals due to the small number of deaths. Results of the Poisson regression models (shown in Table S1) confirmed that in both age groups, sex, time period and country of residence were strongly associated with injury mortality rates, and that overall, England had significantly lower injury mortality rates than Scotland and Northern Ireland.

**Table 1:** Injury mortality rate ratios, rate ratios and rate differences (per 100,000 population) comparing Scotland, Wales and Northern Ireland with England, by age group, sex and time period.

	Boys				Girls			
	Country*				Country*			
28 days-9 years	England	Scotland	Wales	Northern Ireland	England	Scotland	Wales	Northern Ireland
**1980–84**								
Number of deaths/pop.	1,945/14,856,771	325/1,683,945	104/905,791	114/677,152	1,125/14,096,474	180/1,600,463	74/856,068	72/643,497
Mortality rate	13.09 (12.52, 13.69)	19.30 (17.31, 21.52)	11.48 (9.47, 13.91)	16.84 (14.01, 20.22)	7.98 (7.53, 8.46)	11.25 (9.72, 13.02)	8.64 (6.88, 10.86)	11.19 (8.88, 14.10)
Rate ratio cf. England†	–	1.47 (1.31, 1.66)	0.88 (0.72, 1.07)	1.29 (1.07, 1.55)	–	1.41 (1.20, 1.65)	1.08 (0.86, 1.37)	1.40 (1.10, 1.78)
Rate difference cf. England‡	–	6.21 (4.03, 8.39)	−1.61 (−3.89, 0.67)	3.74 (0.60, 6.89)	–	3.27 (1.56, 4.97)	0.66 (−1.36, 2.69)	3.21 (0.58, 5.83)
**1993–97**								
Number of deaths/pop.	998/16,243,952	130/1,634,881	67/963,632	67/661,036	626/15,505,887	83/1,562,430	41/917,082	37/628,382
Mortality rate	6.14 (5.77, 6.54)	7.95 (5.96, 9.44)	6.95 (5.47, 8.83)	10.14 (7.98, 12.88)	4.04 (3.73, 4.37)	5.31 (4.28, 6.59)	4.47 (3.29, 6.07)	5.89 (4.27, 8.13)
Rate ratio cf. England†	–	1.29 (1.08, 1.55)	1.13 (0.88, 1.45)	1.65 (1.29, 2.11)	–	1.32 (1.05, 1.65)	1.11 (0.81, 1.52)	1.46 (1.05, 2.03)
Rate difference cf. England‡	–	1.81 (0.39, 3.23)	0.81 (−0.90, 2.52)	3.99 (1.54, 6.45)	–	1.28 (0.09, 2.46)	0.43 (−0.97, 1.84)	1.85 (−0.07, 3.77)
**2006–10**								
Number of deaths/pop.	480/15,520,377 3	50/1,417,234	29/859,730	18/597,247	325/14,809,639	30/1,352,357	20/815,019	18/566,490
Mortality rate	.09 (2.83, 3.38)	3.52 (2.67, 4.65)	3.37 (2.34, 4.85)	3.01 (1.90, 4.78)	2.19 (1.97, 2.45)	2.22 (1.55, 3.17)	2.45 (1.58, 3.80)	3.18 (2.00, 5.04)
Rate ratio cf. England†	–	1.14 (0.85, 1.53)	1.09 (0.75, 1.59)	0.98 (0.62, 1.55)	–	1.01 (0.70, 1.47)	1.12 (0.71, 1.76)	1.45 (0.90, 2.33)
Rate difference cf. England‡	–	0.44 (−0.58, 1.45)	0.28 (−0.98, 1.54)	−0.08 (−1.50, 1.34)	–	0.02 (−0.81, 0.85)	0.26 (−0.86, 1.36)	0.98 (−0.50, 2.47)
**10–18 years**	**England**	**Scotland**	**Wales**	**Northern Ireland**	**England**	**Scotland**	**Wales**	**Northern Ireland**
**1980–84**								
Number of deaths/pop.	5,057/17,038,295	694/1,990,010	315/1,021,930	241/676,702	1,550/16,169,129	204/1,903,575	102/980,248	65/641,636
Mortality rate	29.68 (28.87, 30.51)	34.87 (32.37, 37.57)	30.82 (27.60, 34.42)	35.61 (31.39, 40.41)	9.59 (9.12, 10.08)	10.72 (9.34, 12.29)	10.41 (8.57, 12.63)	10.13 (7.94, 12.92)
Rate ratio cf. England†	–	1.17 (1.09, 1.27)	1.04 (0.93, 1.16)	1.20 (1.05, 1.37)	–	1.12 (0.97, 1.29)	1.09 (0.89, 1.33)	1.06 (0.82, 1.35)
Rate difference cf. England‡	–	5.19 (2.47, 7.91)	1.14 (−2.36, 4.64)	5.93 (1.36, 10.50)	–	1.13 (−0.42, 2.68)	0.82 (−1.26, 2.89)	0.54 (−1.96, 3.05)
**1993–97**								
Number of deaths/pop.	2,412/13,535,473	359/1,443,819	196/833,699	189/591,838	887/12,968,872	113/1,393,253	52/800,942	54/573,896
Mortality rate	17.82 (17.12, 18.55)	24.86 (22.42, 27.57)	23.51 (20.44, 27.04)	31.91 (27.69, 36.82)	6.84 (6.40, 7.30)	8.11 (6.74, 9.75)	6.49 (4.95, 8.52)	9.41 (7.21, 12.29)
Rate ratio cf. England†	–	1.40 (1.25, 1.56)	1.32 (1.14, 1.53)	1.79 (1.55, 2.08)	–	1.19 (0.97, 1.44)	0.95 (0.72, 1.26)	1.38 (1.05, 1.81)
Rate difference cf. England‡	–	7.04 (4.38, 9.71)	5.69 (2.32, 9.06)	14.11 (9.51, 18.72)	–	1.27 (−0.29, 2.83)	−0.35 (−2.17, 1.47)	2.57 (0.02, 5.12)
**2006–10**								
Number of deaths/pop	1,711/14,841,629	277/1,427,116	140/880,184	120/575,449	611/14,170,350	89/1,357,250	49/841,004	46/547,202
Mortality rate	11.53 (10.99, 12.09)	19.41 (17.25, 21.84)	15.91 (13.48, 18.77)	20.85 (17.44, 24.94)	4.31 (3.98, 4.67)	6.56 (5.33, 8.07)	5.83 (4.40, 7.71)	8.41 (6.30, 11.22)
Rate ratio cf. England†	–	1.68 (1.48, 1.91)	1.38 (1.16, 1.64)	1.81 (1.50, 2.18)	–	1.52 (1.22, 1.90)	1.35 (1.01, 1.81)	1.95 (1.44, 2.63)
Rate difference cf. England‡	–	7.88 (5.53, 10.23)	4.38 (1.69, 7.07)	9.32 (5.55, 13.10)	–	2.25 (0.84, 3.65)	1.51 (−0.15, 3.18)	4.09 (1.64, 6.55)

* Numbers in brackets indicate 95% confidence intervals.

† Rate_Country_/Rate_England._

‡ Rate_Country_- Rate_England._

There was no significant difference in the rate of decline in injury mortality rates between the four countries in children aged 28 days to nine years (LR- test *p* = 0.08 comparing models including sex, country, time period with a model also including a time period:country interaction term). For children aged 10 to 18 years, the addition of a time period:country interaction term improved the fit of the Poisson model (*F*-test *p* = 0.02, Table S1), indicating that observed time trends in mortality rates appear to be significantly different in the four UK countries. As shown in Table 1, for older boys the trend is towards increasing disparity, with Scotland, Wales and Northern Ireland experiencing increasingly higher mortality over time compared with England. Increasing relative differences in mortality rates over time between England and Northern Ireland are also apparent for girls aged 10 to 18 years.

If all countries had had the same mortality rates as England for 10 to 18 year old boys, there would have been 113 fewer deaths in Scotland, 39 in Wales and 54 in Northern Ireland during the period 2006 to 2010 in this age group. For girls aged 10 to 18 years, there would have been 31 fewer deaths in Scotland and 23 fewer in Northern Ireland if they had experienced the same mortality rates as English girls of the same age. The 95% confidence interval for the rate difference comparing mortality rates in Wales and England for 10 to 18 year old girls included zero. 95% confidence intervals for between-country risk differences included zero for younger children in all countries.

**Figure 3:**
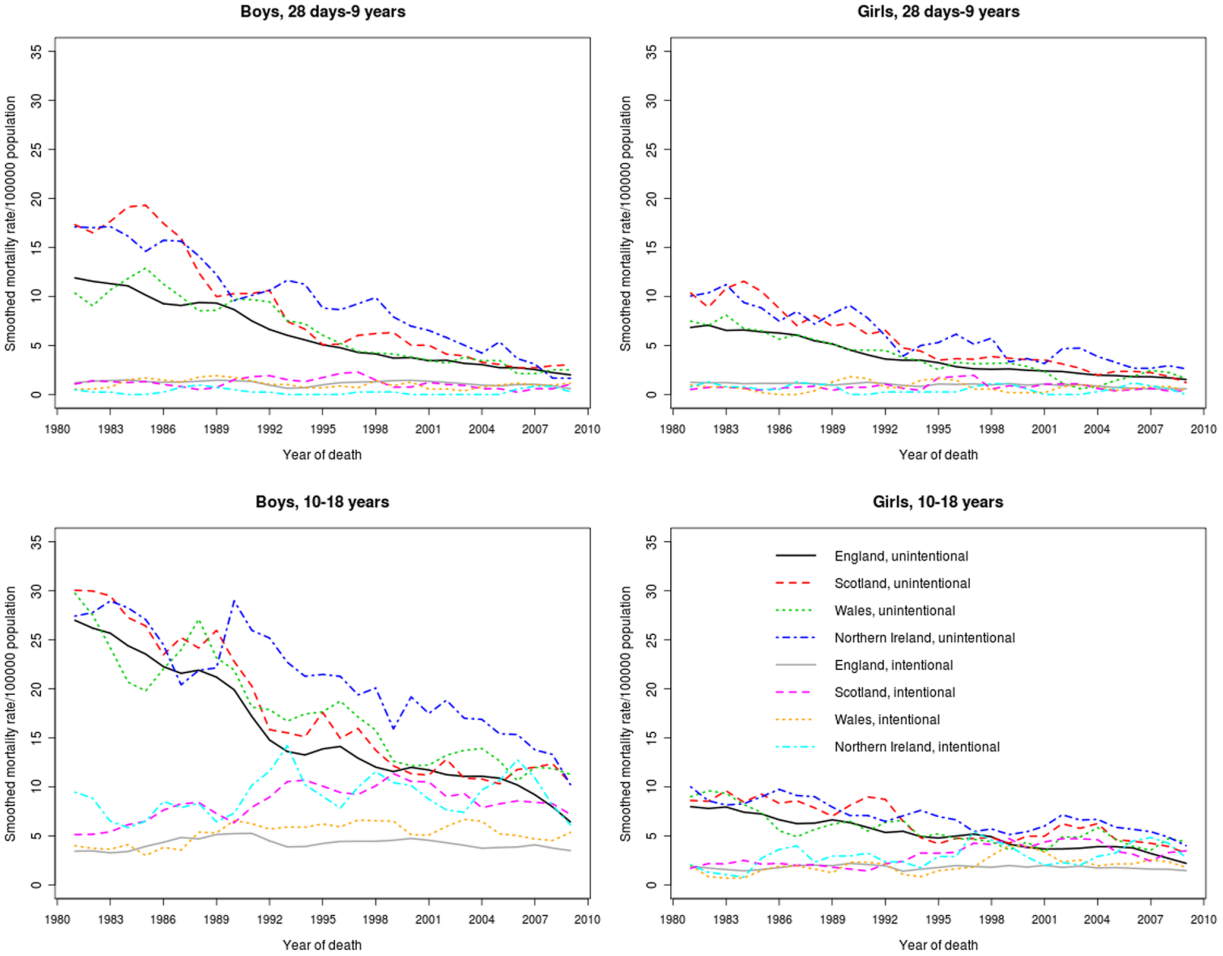
Injury mortality rates by year of death, gender, country and cause of injury by age group 1980–2010 (three-year moving averages). Note the three-year average rates are plotted on the central year.

Figure 3 and Table 2 shows that the decline in injury mortality in all four countries was accounted for by a decrease in unintentional injury deaths, with similar declines in traffic accidents and other accidents within the two age groups. There has been no decline since 1980 in intentional causes of injury in any country for children aged 10 to 18 years. A decline was observed in England for younger children. In the latest period, intentional injuries accounted for 34.1% (767/2,248) of injury deaths among boys aged 10 to 18 years and 37.7% (300/795) of injury deaths among girls in the same age group across the four UK countries.

**Table 2:** Mortality rates (per 100,000 population) with 95% confidence intervals by country, age group, time period and type of injury (due to transport accidents, other accidents or intentional).

	Transport accidents			
	Country			
28 days-9 years	England	Scotland	Wales	Northern Ireland
**1980–84**				
Number of deaths/pop.	1,147/28,953,245	192/3,284,408	63/1,761,859	100/1,320,649
Rate/100000 pop (95% CI)	3.96 (3.73, 4.20)	5.85 (5.07, 6.73)	3.58 (2.79, 4.58)	7.57 (6.22, 9.21)
**1993–97**				
Number of deaths/pop.	–	77/3,197,311	–	61/1,289,418
Rate/100000 pop (95% CI)		2.41 (1.93, 3.01)		4.73 (3.68, 6.08)
**2006–10**				
Number of deaths/pop.	180/30,330,016	32/2,769,591	12/1,674,749	16/1,163,737
Rate/100000 pop (95% CI)	0.59 (0.51, 0.69)	1.16 (0.82, 1.63)	0.72 (0.41, 1.26)	1.37 (0.84, 2.24)
**10–18 years**	**England**	**Scotland**	**Wales**	**Northern Ireland**
**1980–84**				
Number of deaths/pop.	4,324/33,207,424	534/3,893,585	224/2,002,178	183/1,318,338
Rate/100000 pop (95% CI)	13.02 (12.64, 13.42)	13.71 (12.60, 14.93)	11.19 (9.82, 12.75)	13.88 (12.01, 16.05)
**1993–97**				
Number of deaths/pop.	–	212/2,837,072	–	115/1,165,734
Rate/100000 pop (95% CI)		7.47 (6.53, 8.55)		9.87 (8.22, 11.84)
**2006–10**				
Number of deaths/pop	1,124/29,011,979	166/2,784,366	76/1,721,188	69/1,122,651
Rate/100000 pop (95% CI)	3.87 (3.65, 4.11)	5.96 (5.12, 6.94)	4.42 (3.53, 5.53)	6.15 (4.83, 7.78)
	**Other unintentional injuries**			
**28 days-9 years**	**England**	**Scotland**	**Wales**	**Northern Ireland**
**1980–84**				
Number of deaths/pop.	1,562/28,953,245	282/3,284,408	101/1,761,859	79/1,320,649
Rate/100000 pop (95% CI)	5.39 (5.13, 5.67)	8.59 (7.64, 9.65)	5.73 (4.72, 6.97)	5.98 (4.80, 7.46)
**1993–97**				
Number of deaths/pop.	–	85/3,197,311	–	41/1,289,418
Rate/100000 pop (95% CI)		2.66 (2.15, 3.29)		3.18 (2.34, 4.32)
**2006–10**				
Number of deaths/pop.	414/30,330,016	33/2,769,591	22/1,674,749	14/1,163,737
Rate/100000 pop (95% CI)	1.36 (1.24, 1.50)	1.19 (0.85, 1.68)	1.31 (0.86, 2.00)	1.20 (0.71, 2.03)
**10–18 years**	**England**	**Scotland**	**Wales**	**Northern Ireland**
**1980–84**				
Number of deaths/pop.	1,429/33,207,424	219/3,893,585	136/2,002,178	64/1,318,338
Rate/100000 pop (95% CI)	4.30 (4.09, 4.53)	5.62 (4.93, 6.42)	6.79 (5.74, 8.04)	4.85 (3.80, 6.20)
**1993–97**				
Number of deaths/pop.	–	74/2,837,072	–	54/1,165,734
Rate/100000 pop (95% CI)		2.61 (2.08, 3.28)		4.63 (3.55, 6.05)
**2006–10**				
Number of deaths/pop	424/29,011,979	42/2,784,366	50/1,721,188	25/1,122,651
Rate/100000 pop (95% CI)	1.46 (1.33, 1.61)	1.51 (1.11, 2.04)	2.90 (2.20, 3.83)	2.23 (1.50, 3.30)
	**Intentional injuries**			
**28 days-9 years**	**England**	**Scotland**	**Wales**	**Northern Ireland**
**1980–84**				
Number of deaths/pop.	361/28,953,245	31/3,284,408	14/1,761,859	7/1,320,649
Rate/100000 pop (95% CI)	1.25 (1.12, 1.38)	0.94 (0.66, 1.34)	0.79 (0.47, 1.34)	0.53 (0.25, 1.11)
**1993–97**				
Number of deaths/pop.	293/31,749,839	51/3,197,311	19/1,880,714	−/1,289,418*
Rate/100000 pop (95% CI)	0.92 (0.82, 1.03)	1.60 (1.21, 2.10)	1.01 (0.64, 1.58)	
**2006–10**				
Number of deaths/pop.	211/30,330,016	15/2,769,591	15/1,674,749	6/1,163,737
Rate/100000 pop (95% CI)	0.70 (0.61, 0.80)	0.54 (0.33, 0.90)	0.90 (0.54, 1.49)	0.52 (0.23, 1.15)
**10–18 years**	**England**	**Scotland**	**Wales**	**Northern Ireland**
**1980–84**				
Number of deaths/pop.	854/33,207,424	145/3,893,585	57/2,002,178	59/1,318,338
Rate/100000 pop (95% CI)	2.57 (2.40, 2.75)	3.72 (3.16, 4.38)	2.85 (2.20, 3.69)	4.48 (3.47, 5.78)
**1993–97**				
Number of deaths/pop.	789/26,504,345	186/2,837,072	59/1,634,641	74/1,165,734
Rate/100000 pop (95% CI)	2.98 (2.78, 3.19)	6.56 (5.68, 7.57)	3.61 (2.80, 4.66)	6.35 (5.05, 7.97)
**2006–10**				
Number of deaths/pop	774/29,011,979	158/2,784,366	63/1,721,188	72/1,122,651
Rate/100000 pop (95% CI)	2.67 (2.549, 2.86)	5.67 (4.86, 6.60)	3.66 (2.86, 4.69)	6.41 (5.09, 8.08)

* Number of deaths is ≤5 in this group and the count and rate are therefore suppressed to avoid disclosure.

Note that mortality rates according to accident type for England and Wales in 1993–97 were not calculated due to a large number of deaths from traffic accidents were coded as unspecified other accidents in this period.

## Discussion

Rates of child deaths due to injury have declined since 1980 in all four UK countries by age and sex but Scotland, Wales and Northern Ireland continue to experience higher mortality rates than England. Differences between England and the other UK countries have increased for boys aged 10 to 18 years. There has been little or no decrease in intentional injury deaths since the 1980s.

Similar declines in deaths due to injury have been observed in other European countries [15], Australia [16] and the United States [17], although both the Unites States and Australia have historically had higher mortality from injury than the UK [18]. Increased use of safety measures such as traffic calming, bike helmets, smoke alarms and swimming classes have collectively been linked to the decline in injury deaths in children, although the effects of individual interventions have not been evaluated [19]. Less exposure to traffic as children use cars rather than walk has also been associated with a decline in road traffic accidents [20].

An important advantage of using data from death certificates is the universal coverage of all deaths in each country. We did not have access to indicators of socio-economic deprivation and parental educational attainment for this study. These risk factors have been associated with child mortality [5], [6], [8], [21], and may explain some of the inter-country differences observed in this study.

A limitation of death certification data is the substantial delays in reporting injury deaths in England, Wales and Northern Ireland. We allowed for this by including deaths registered up to 18 months after the end of the study period; further adjustment for reporting delays made little difference to the rates. Despite this, the last three to five years of the study period should still be viewed as being subject to increased uncertainty compared with earlier years.

We were able to include revised mid-year population estimates based on the 2011 Census for England and Wales; revised population estimates were not available for Scotland and Northern Ireland. A crude comparison of the number of children in Scotland and Northern Ireland in the 2011 Census and unrevised population estimates showed a shortfall of 2–4% among children aged 10 years and over. Even allowing for a 4% shortfall in these populations, estimated injury mortality rates would not change significantly when the revised population estimates become available.

National socioeconomic indicators do not show a consistent pattern that can explain the observed trends in injury mortality rates, for which England has experienced the lowest rates in both boys and girls since 1980. For example, relative child poverty after housing costs has been lower in Scotland and Northern Ireland than in England during the last half of the study period [22]. Whilst employment rates have been higher in England than in Wales and Northern Ireland since 2004, employment rates have been similar, or higher, in Scotland [23]. Similarly, educational achievement appears to be highest in Scotland and lowest in Northern Ireland; in 2005 the proportion of children with no educational qualifications was 8.3% in Scotland and 19.9% in Northern Ireland [24]. Prevalence of risky health behaviours that might increase the risk of death from injuries do not appear to be consistent with higher rates of injury deaths in Northern Ireland and Scotland compared with England. The latest available data (from 1997/98) indicate similar levels of daily smoking prevalence among 15-year olds in the four UK countries, whereas the proportion of 15 year olds who drink alcohol weekly was lower in Northern Ireland than in England, Scotland and Wales [25]. In any case these indicators would only provide evidence of an ecological association and no causal link.

A better understanding of factors that account for the differences between the four UK countries in rates of injury deaths requires linkage of death certificate data to individual level information on variables such as parental employment, receipt of benefits, educational attainment, social care, crime involvement and hospital or primary care use. Linkage of death certificates to other nationally collected datasets such as hospital inpatient data, school records or census datasets have already been exploited by Scandinavian research groups and show associations between child mortality and parental educational attainment [8], adoption and foster care status [26] and parental marital status [27]. Linkage of such data is theoretically feasible in all four UK countries provided the legislative framework can be put in place [28].

We found increased rates of death due to injury in Scotland, Wales, and Northern Ireland compared to England, particularly among older boys. This group stands to benefit most from policies and programmes to reduce injury deaths. Linkage of death certificates to other data reflecting social, educational and health determinants of childhood injury deaths would improve understanding of the reasons for variation across the UK and assist in targeting preventive strategies.

## Supporting Information

Figure S1 **Proportion of deaths reported (p_i_) according to delay between occurrence and registration, for children aged 15–18 where the death was due to an injury.** Note that while the x-axis is labelled in years, the delay distribution was calculated by month.
(TIF)

Table S1 **Estimated injury mortality rate ratios from Poisson regression models.**
(DOCX)

Text S1 **Methods used to adjust counts of deaths for reporting delay.**
(DOCX)
