# Correction: Child Deaths Due to Injury in the Four UK Countries: A Time Trends Study from 1980 to 2010

**DOI:** 10.1371/annotation/9efc9262-f6e2-4b9c-823a-93986608d8a3

**Published:** 2014-01-02

**Authors:** Pia Hardelid, Jonathan Davey, Nirupa Dattani, Ruth Gilbert, the Working Group of the Research and Policy Directorate of the Royal College of Paediatrics and Child Health

There is an error in the Supporting Information file (Text S1) in the first paragraph. The correct sentence is “An example of pi is demonstrated in Figure S1 for England and Wales and Northern Ireland, for deaths in children aged 15 to 18 years where the death was due to an injury.”

There was a formatting issue in Table 2 the PDF version of this article. The table continues on page 6 and should read “Intentional Injuries” not “Transport accidents.” Please view the correct Table 2 on the PLOS ONE website.

