# Supplementary figures and images for: Child Deaths Due to Injury in the Four UK Countries: A Time Trends Study from 1980 to 2010
Source: PLoS One. 2013 Jul 10;8(7):e68323. doi: 10.1371/journal.pone.0068323 (PMC3707924; doi:10.1371/journal.pone.0068323)

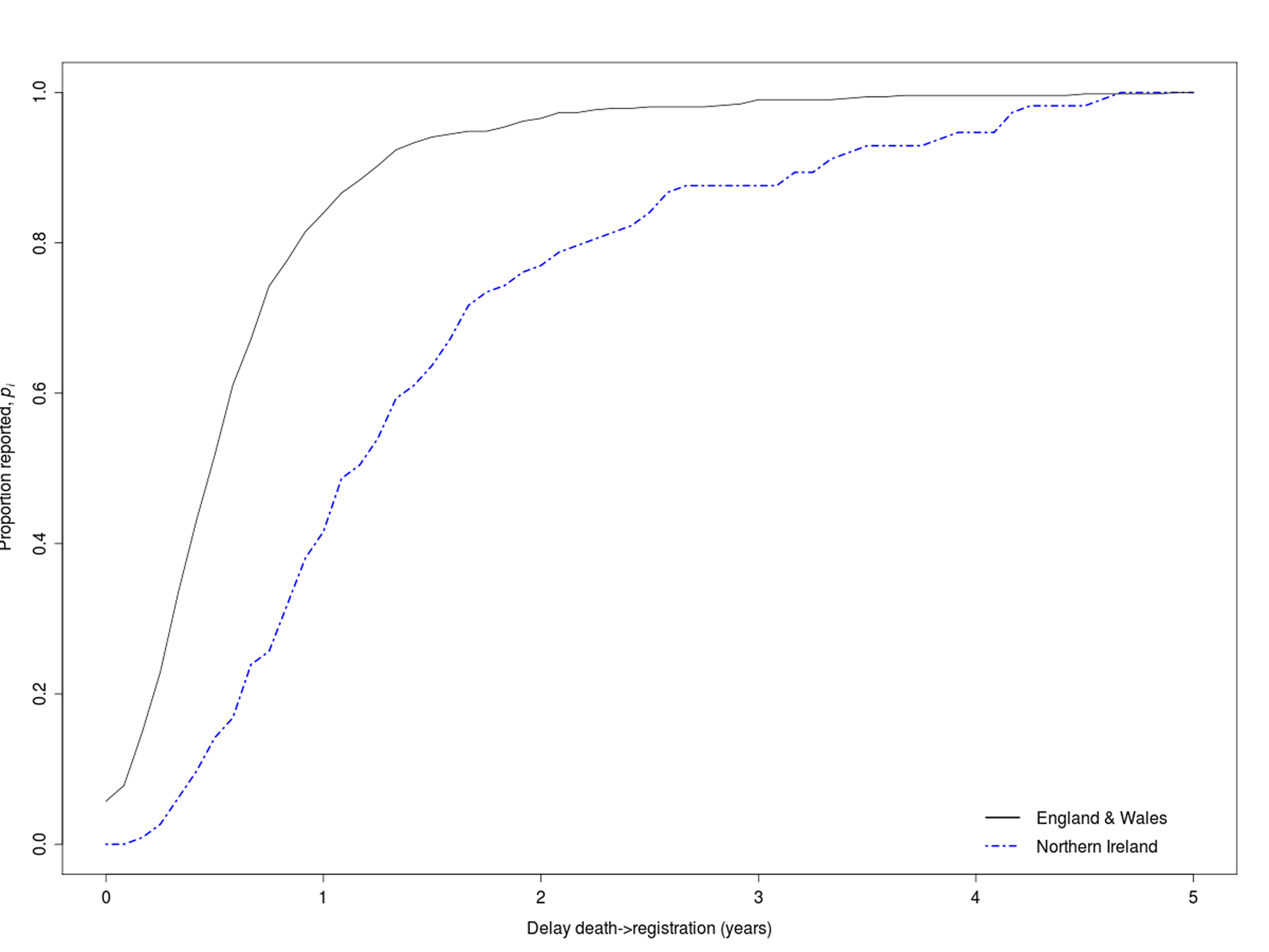

Supplement: Figure S1 — Proportion of deaths reported (pi) according to delay between occurrence and registration, for children aged 15–18 where the death was due to an injury. Note that while the x-axis is labelled in years, the delay distribution was calculated by month. (TIF) [file pone.0068323.s001.tif]
