# Supplementary material for: Child Deaths Due to Injury in the Four UK Countries: A Time Trends Study from 1980 to 2010
Source: PLoS One. 2013 Jul 10;8(7):e68323. doi: 10.1371/journal.pone.0068323 (PMC3707924; doi:10.1371/journal.pone.0068323)
Supplement: Table S1 — Estimated injury mortality rate ratios from Poisson regression models. (DOCX) [file pone.0068323.s002.docx]

| **28 days – 9 years^†^** | **Mortality rate ratio** |
| --- | --- |
| **Country**  England  Scotland  Wales  Northern Ireland | 1  1.36 (1.26, 1.47)^***^  1.02 (0.91, 1.14)  1.38 (1.23, 1.54)^***^ |
| **Sex**  Male  Female | 1  0.64 (0.61, 0.67)^***^ |
| **Time period**  1980-84  1993-97  2006-2010 | 1  0.48 (0.46, 0.51)^***^  0.24 (0.23, 0.26)^***^ |
| **10-18 years^‡^** | **Mortality rate ratio** |
| **Sex**  Male  Female | 1  0.34 (0.32, 0.36)^***^ |
| **Country (1980-1984)**  England  Scotland  Wales  Northern Ireland | 1  1.16 (1.05, 1.29)^* *^  1.05 (0.91, 1.21)  1.17 (0.99, 1.38) |
| **Country (1993-97)**  England  Scotland  Wales  Northern Ireland | 1  1.34 (1.16, 1.54) ^***^  1.22 (1.01, 1.47) ^*^  1.68 (1.39, 2.03) ^***^ |
| **Country (2006-2010)**  England  Scotland  Wales  Northern Ireland | 1  1.64 (1.40, 1.93) ^***^  1.37 (1.10, 1.71) ^**^  1.85 (1.46, 2.33) ^***^ |

*****Wald test *p*<0.05 **Wald test *p*<0.01 ***Wald test *p*<0.001

**^†^**LR-test *p*-values values comparing deviance of model with all three covariates compared to model excluding variable: sex *p*<0.001, time period: *p*<0.001, country: *p*<0.001

**^‡^**Model for 10-18 year old children fitted using a quasi-likelihood method to take into account overdispersion. *F*-test *p* values comparing deviance of model with all three covariates compared to model excluding variable: sex *p*<0.001, time period: *p*<0.001, country: *p*<0.001, time:country interaction (cf. model with no interaction term) *p*=0.02

Table S1. Estimated injury mortality rate ratios from Poisson regression models
