# Supplementary material for: Child Deaths Due to Injury in the Four UK Countries: A Time Trends Study from 1980 to 2010
Source: PLoS One. 2013 Jul 10;8(7):e68323. doi: 10.1371/journal.pone.0068323 (PMC3707924; doi:10.1371/journal.pone.0068323)
Supplement: Text S1 — Methods used to adjust counts of deaths for reporting delay. (DOCX) [file pone.0068323.s003.docx]

**Supporting Information: Methods used to adjust counts of deaths for reporting delay**

The methods used to adjust for reporting delay in this study are similar to those presented by Hardelid et al[1] and Green et al[2]. The observed proportion of deaths registered within *i* months of death *p_i_*, was estimated according to age group (28 days-364 days, one to four, five to nine, 10 to 14 and 15 to 18 years), gender and underlying cause of death, using deaths registered between 2006 and 2010 for England and Wales, and 1997 and 2010 for Northern Ireland. *p_i_* therefore represents the proportion of deaths reported with a particular number of months’ delay. An example of *p_i_* is demonstrated in Figure S1 for England and Wales and Northern Ireland, for deaths in children aged 10 to 18 years where the death was due to an injury. The proportion of deaths reported by month since occurrence was assumed to be the same in England and Wales since they share death reporting systems.

We assumed all deaths had been reported within three years for England and Wales, and five years in Northern Ireland. The adjusted count of deaths occurring in a particular month *i* (*adj_count_i_*) can be estimated as:

${adj\_count}_{i}={{obs\_count}_{i}}/{p_{i}}$, Eq. 1

where *obs_count_i_* is the observed count of deaths. The monthly adjusted counts were then aggregated into years and time periods. To adjust confidence intervals for the increased uncertainty incurred by reporting delay, standard errors were inflated using the following method

$95\%CI=\exp\left( {log}_{e}\left( \lambda_{adj} \right)\pm z_{0.975}\frac{1}{\sqrt{obs\_count}} \right)$ Eq. 2

Where *λ_adj_* is the unadjusted mortality rate, *z* is the area under the curve of the standard normal distribution, and *obs_count* is the unadjusted count. To calculate confidence intervals for a difference in rate ratios based on adjusted counts, the following formula was used:

${95\%CI}_{IRR}=\exp\left( {log}_{e}\left( {IRR}_{adj} \right)\pm\sqrt{\left( \frac{1}{{obs\_count}_{1}}+\frac{1}{{obs\_count}_{2}} \right)} \right)$ Eq. 3

Where *IRR_adj_* is the adjusted mortality rate ratio, and *obs_count_1_* and *obs_count_2_* are the unadjusted counts for country 1 and country 2.

Similarly, confidence intervals for the difference in rates were estimated as:

${95\%CI}_{RD}={RD}_{adj}\pm\sqrt{\left( \frac{{adj\_count}_{1}}{{py}_{1}^{2}}\times\frac{1}{p_{1}}+\frac{{adj\_count}_{2}}{{py}_{2}^{2}}\times\frac{1}{p_{2}} \right)}$ Eq. 4

where *RD_adj_* is the adjusted rate difference, *p_1_* and *p_2_* are the estimated proportion of deaths reported (*p*=*obs_count*/*adj_count*) and *py_1_* and *py_2_* are the person years for country 1 and 2 respectively.

**References**

1. Hardelid P, Andrews N, Pebody R (2011) Excess mortality monitoring in England and Wales during the influenza A(H1N1) 2009 pandemic. Epidemiol Infect 139: 1431-1439.

2. Green HK, Andrews NJ, Bickler G, Pebody RG (2012) Rapid estimation of excess mortality: nowcasting during the heatwave alert in England and Wales in June 2011. J Epidemiol Community Health 66: 866-868.
